# Cognitive decline, sensory impairment, and the use of audio-visual aids by long-term care facility residents

**DOI:** 10.1186/s12877-022-02895-x

**Published:** 2022-03-16

**Authors:** Rick Yiu Cho Kwan, Chi Wai Kwan, Patrick Pui Kin Kor, Iris Chi

**Affiliations:** 1 School of Nursing, Tung Wah College, Homantin Kowloon, Hong Kong, Hong Kong; 2 Department of Statistics and Actuarial Science, The University of Hong Kong, Pok Fu Lam, Hong Kong, Hong Kong; 3 School of Nursing, The Hong Kong Polytechnic University, Hung Hom, Hong Kong, Hong Kong; 4 Suzanne Dworak-Peck School of Social Work, University of Southern California, 669 W. 34th Street, Los Angeles, CA 90089-0411 USA

**Keywords:** Cognitive decline, Hearing aid, Hearing impairment, Visual aids, Visual impairment

## Abstract

**Background:**

Hearing and vision impairments and the use of audio-visual aids are associated with cognitive decline in community-dwelling older people, but effects in long-term care facilities (LFCF) are unclear. We hypothesize that visual and hearing impairment are associated with cognitive decline and these relationships are mediated by using visual and hearing aids.

**Methods:**

Secondary data analysis of a longitudinal study was conducted in the 7 government-subsidized LTCF operated by one of the largest non-governmental organizations in Hong Kong using data between 2005 and 2016. Eligible residents were ≥ 60 years of age without severe cognitive impairment at baseline who had stayed in the facilities for more than 3 years. All variables were measured by using the Minimum Data Set-Resident Assessment Instrument Version 2.0, Hong Kong version. The outcome was cognitive decline. Predictors were visual and hearing impairments. Mediators were the use of visual and hearing aids. General linear models were employed to test the hypotheses.

**Results:**

Results for 2,233 residents were analyzed, with a mean age of 82.1 ± 8.2 years and a mean follow-up period of 4.4 ± 0.8 years. Results showed that those who had visual impairment (*p* = 0.004) and hearing impairments (*p *= 0.022) had a higher risk of cognitive decline. Using hearing aids (coefficient = 0.0186, *p* < 0.05) positively mediates the effect of hearing impairment on cognitive decline. Using visual aids (coefficient = -0.0881, *p* < 0.05) negatively mediates the effects of visual impairment on cognitive decline.

**Conclusion:**

In LTCF, hearing and visual impairments are associated with a higher risk of cognitive decline. Hearing aids often-users were associated with a higher risk of cognitive decline. LTCF residents with visual impairment did not use visual aids. Use of visual aids demonstrated potential effects in slowing cognitive decline. A future study with a larger and more diverse sample with attention to quality of devices is proposed to confirm its effects.

## Introduction

Cognitive decline refers to a longitudinal decline in cognitive function [1]. Cognitive decline is mostly explained by ageing, which begins relatively early in adulthood but accelerates after the age of 60 [2]. Age-associated cognitive decline can be a normal manifestation of neurodegeneration throughout the process of ageing [3]; it is difficult to differentiate between pathological and non-pathological cognitive decline as the age-associated cognitive decline is a result of the synergistic effect of pathological (e.g., accumulation of β-amyloid) and non-pathological (e.g., brain volume loss) causes and it thus varies among individuals [4–6]. A faster rate of cognitive decline at the pre-clinical stage (e.g., subjective cognitive decline) is not only associated with an increased risk of dementia [7], but is also strongly related to mortality in people with Alzheimer’s dementia [8]. Therefore, cognitive decline is an important symptom to track in assessing health and planning care.

Hearing impairment refers to a reduction in hearing sensitivity that causes difficulty in daily living [9]. It is classified into two types: conductive (e.g., cerumen impaction) or sensorineural (e.g., age-related changes) [10]. In a population-based cohort study, the prevalence of hearing impairment in older people in some LTCFs was reported to be 23.3% in Gujo City, Gifu Prefecture, Japan, [11] and the prevalence increases with age. [12] Visual impairment describes a loss of sight causing difficulty in daily living and is commonly caused by age-related conditions (e.g., presbyopia, cataract) [13, 14]. The prevalence rate of visual impairment in older people in LTCFs was reported to be 14.4% in Gujo City, Gifu Prefecture, Japan, which also increases with age [11]. We found no reports of prevalence specific to Hong Kong.

Hearing aids are common instruments available to older people to improve their audibility, thereby boosting their social interactions and improving their quality of life [15]. However, the use of hearing aids by older people with hearing impairment is suboptimal [16]. Specifically, in LTCFs, the prevalence of the use of hearing aids is 11.5–16.8% [17]. Underdetection of hearing loss and underuse of hearing aids are common [18]. Investigators have reported that 70–85% of the older people with hearing impairment did not have prescribed hearing aids and 45% of those who were prescribed hearing aids were not using them [19]. However, there is a lack of data on the extent of use of hearing aids as reported in a systematic review [20]. The use of hearing aids by older people in long-term settings is particularly under-reported. Reasons for not using hearing aids vary from an underestimation of hearing loss by the individual, complaints of poor fit and discomfort, cost, to inappropriate referrals [21, 22]. Visual aids, also known as optical aids, are commonly prescribed (62%) and used in daily life (54.8%) by older people with visual impairment [23]. Unlike hearing aids, the majority of older people with visual impairment who are prescribed visual aids use them regularly [23]. Previous studies indicate that vision rehabilitation including the use of visual aids could improve the clinical and functional ability outcomes and demonstrate the potential effects on mood and health-related quality of life [24].

Literature indicates that hearing impairment significantly predicts cognitive decline, mild cognitive impairment and Alzheimer’s dementia in prospective cohort studies [25–27]; visual impairment is associated with the risk of dementia and predicts cognitive decline [28, 29]. Cognitive decline that occurred in long-term care facility (LTCF) residents with dual sensory impairment (i.e., the co-existence of visual and hearing impairment) was faster than in those with only one impairment and those without any sensory impairment [30]. Observational and questionnaire studies have demonstrated that the use of hearing aids slowed cognitive decline in older people [31–34]. However, the protective effect of using hearing aids was not observed in individuals with Alzheimer’s dementia [35].

Although links between cognitive decline, hearing and vision impairment, and hearing aid use were reported in studies, some important knowledge is still missing. First, observations of the promising effect of hearing aids on cognitive function were mostly for community-dwelling older adults. One recent article reported on hearing loss and its impact on residents in LTCFs; however, investigators only reported the effects of cerumen removal on cognitive function [36]. Therefore, the effect of hearing aids on cognitive function in LTCFs is unknown. Second, there are few reports of the effect of visual aid use on cognitive decline [37, 38] in long term care dwelling older adults. The Cascade hypothesis theorizes that a cascade starts from sensory loss, communication failure, and resulting in limited social integration, to a decrease in socialization. These detriments related to sensory loss can cascade directly or indirectly to cognitive impairment [39]. In LTCFs, older people are mostly confined to indoor settings with simpler socialization compared with those dwelling in the community. It is unclear whether effective audio-visual aids could slow cognitive decline through the rectification of sensory losses in LTCFs. It is crucial to clarify this point as such evidence can be used to formulate policies on health screenings and adherence monitoring if the use of audio-visual aids eases cognitive decline in older people in LTCFs.

## Objectives

The objectives of this study are to examine 1) the association between sensory impairment and cognitive decline and 2) the mediation effect of the use of audio-visual aids between sensory impairment and cognitive decline in LTCFs. We hypothesize that:

1. Sensory impairment is associated with cognitive decline, and
2. The use of audio-visual aids mediates the effect of the sensory impairment on cognitive decline.

## Methods

### Study design

This was a secondary data analysis from the Hong Kong Longitudinal Study on Long-Term Care Facility Residents, in which all residents in 7 LTCFs were repeatedly assessed. The exact period between two repeated assessments could not be precisely controlled because of the availability of the residents (e.g., hospitalization) and the availability of certified assessors. The period of two repeated assessments of each resident varied but ranged from 6 to 12 months. In that project, health data were routinely collected from a cohort of Chinese long-term care residents in Hong Kong to review and improve clinical practices in LTCFs. The data were collected between January 2005 and December 2016.

The advantage of using regularly collected clinical data for analysis is that it allows dynamic relationships between variables to be examined over time. Secondary data analysis refers to an analysis of data that are collected by someone else for another primary purpose [40]. We followed the guideline for The REporting of studies Conducted using Observational Routinely-collected health Data (RECORD) Statement [41].

### Setting

The study was conducted in the 7 government-subsidized LTCFs, also known as Residential Care Services for the Elderly, operated by one of the largest non-governmental organizations in Hong Kong. Their services include residential care, meals, personal care and limited nursing care for elders who suffer from poor health or physical/mild mental disabilities with a deficiency in daily living activities but who are mentally suitable for a communal living [42].

### Participants

Only residents in the dataset who fulfilled the following eligibility criteria were selected for analysis:

#### Inclusion criteria

1. Older people as defined by WHO at age ≥ 60 years at baseline [43],

#### Exclusion criteria

1. Those who had no follow-up data,
2. Those whose baseline cognitive impairment is severe, as defined by a Cognitive Performance Scale score of ≥ 5 (i.e., possible score range = 0–6) [44], as there is limited room for them to decline further, and
3. Those who have resided in the LTCF for less than 3 years, because the extent of the cognitive decline in 3 years in people without dementia and with early dementia is relatively small (i.e., 0–2 MMSE points decline/year) [45]. The Cognitive Performance Scale is less likely to be sensitive enough to identify minute cognitive decline because each Cognitive Performance Scale point difference varies from 0.8 to 6.3 MMSE points [46].

### Data sources and measurement

The Minimum Data Set-Resident Assessment Instrument Version 2.0 (MDS-RAI 2.0), Hong Kong version, was utilized as the measurement tool [44, 47]. The instrument is a comprehensive tool measuring LTCF residents’ care needs with 22 sections (e.g., cognitive patterns, communication/hearing patterns, vision patterns, and disease diagnosis). For this report, the data set for three sub-scales was used: Cognitive Performance, Hearing, and Vision scales were used. The assessment drew on multiple data sources, which included direct questioning of care recipients and caregivers, observation of care recipients in the long-term care environment, and a review of related documents such as medical records. Various trained professionals (nurses, social workers, occupational therapists, and physiotherapists) collected the data following the standardized MDS-RAI 2.0 Users’ Manual [48]. Nurses monitored the assessment process. The organization conducted in-house standardized training for each assessor. The MDS has good criterion validity on the three sub-scales and good reliability in 80–90% of items [49]. Cognitive function was measured using the subscale Cognitive Performance Scale [44], a hierarchical scale assessing cognitive function specifically in five areas: short-term memory, cognitive skills for daily decision-making, the ability to make oneself understood, comatose status, and dependence on eating [44]. Scores range from 0 to 6; a higher score entails poorer cognitive function. Cognitive Performance Scale has good inter-rater reliability (Spearman *ρ* = 0.85) [50], as well as good agreement with MMSE (*r* = -0.863, *p* < 0.001) good criterion validity with MMSE to identify cognitive impairment (sensitivity = 0.90–0.94, specificity = 0.85–0.95) [50].

The Vision and Hearing scale have good accuracy to identify hearing loss (sensitivity = 0.97, specificity = 0.93) and vision loss (sensitivity = 1.0, specificity = 0.93) compared against performance-based measures [51], as well as good inter-rater reliability for vision (mean Kappa = 0.85) and hearing (mean Kappa = 0.83) [52]. Additional variables for hearing impairment, vision impairment, dual impairment, use of visual and audio aids were developed by recoding relevant single items scores from the MDS as described below.

### Variables

#### Demographic and related clinical profile

Age, gender, cognitive function at baseline, follow-up year, and related comorbidities were collected to describe residents’ demographic and clinical profiles.

#### Predictors

Hearing impairment was measured by the item entitled “Hearing” in the section on Hearing Patterns in the MDS-RAI 2.0. Residents’ hearing impairment was quantified to a score ranging from 0 to 3, which was in turn re-coded as a dichotomous variable. No Impairment described being able to adequately hear normal talk, TV, and phone (i.e., Hearing score = 0); Impairment (i.e., Hearing score = 1–3) ranged from having minimal difficulty hearing (e.g., having difficulty hearing when not in a quiet setting) to being highly impaired (e.g., absence of useful hearing).

Visual impairment was measured by the item entitled “Vision” in the section on Vision Patterns in the MDS-RAI 2.0. Residents’ visual impairment was quantified to a score ranging from 0 to 4, which was then re-coded as a dichotomous variable. No Impairment (i.e., Vision score = 0–1) referred to seeing fine details adequately, including regular or large print in newspapers or books. Impairment (i.e., Vision score = 2–4) either indicated moderately impaired vision (e.g., not able to see newspaper headlines but able to identify objects) or severe impairment (e.g., no vision or only able to see light, colours, or shapes).

Dual sensory impairment was measured by combining the re-coded hearing impairment and visual impairment scores. Residents were categorized as follows: 1) no impairment (i.e., no hearing or visual impairment), 2) one sensory impairment (i.e., having either hearing or visual impairment), and 3) dual impairment (i.e., being both audibly and visually impaired).

#### Mediators

The hearing aid use pattern was measured by the item entitled “Communication Devices” in the section on Hearing Patterns in the MDS-RAI 2.0. Residents’ use of hearing aids fell into one of these four categories: a) hearing aid present and used, b) hearing aid present and not used regularly, c) other receptive communication techniques used, and d) none of the above. The use of hearing aids was re-coded into a 3-point categorical variable: 1) hearing aid present and used (i.e., category a), 2) hearing aid present and not used regularly (i.e., category b), and 3) having no / not using hearing aids (i.e., combining categories c and d).

Visual aid use was measured by the item entitled “Visual Appliances” in the section on Visual Patterns in the MDS-RAI 2.0. It is a dichotomous variable. Visual aid use denoted the use of glasses, contact lenses, or magnifying glasses. Not using visual aids referred to not observed with those devices.

#### Outcome

Cognitive decline was captured by the change in cognitive function from baseline to the last time point of observation (i.e., T0-T1). A higher score indicates more severe cognitive decline.

#### Confounders

Residents’ age and gender were measured at baseline in the database. Cognitive function at baseline, which is associated with the subsequent cognitive decline [53], and co-morbidities, including diabetes mellitus (DM), hypertension (HT), stroke, and dementia [54–56], known to confound the effect of the sensory impairment on the cognitive decline were controlled.

### Statistical methods

IBM SPSS Statistics 25 was utilized to conduct the statistical analysis. Mean with standard deviation and frequency with percentage were used to describe residents’ profiles and related variables in this study. To test hypothesis #1, a univariate general linear model was employed, where cognitive decline served as the dependent variable and hearing impairment, visual impairment, and dual sensory impairment served as the independent variables. To test hypothesis #2, the test of linear moderated mediation using PROCESS macro for SPSS was employed [57]. The dependent variable was cognitive decline, independent variables were hearing impairment and visual impairment, and the mediators were the use of hearing aids and visual aids. All models were adjusted for known confounders (i.e., age, gender, baseline cognitive function, DM, HT, dementia, and stroke), and a two-tailed alpha level of 0.05 was used in all analyses. The estimates of the effects of the predictors were reported using either F-statistics or the estimated marginal mean difference of the outcome (i.e., the CPS change score between categories).

### Data access and cleaning methods

The authors are team members of the “Well-being and Associated Factors of Vulnerable Populations in Long-term Care in Hong Kong” project, entailing our direct access to the database. After extracting data according to the eligibility criteria, we excluded participants with missing data in any variables involved in this analysis.

## Results

### Study sample

**Fig. 1:**
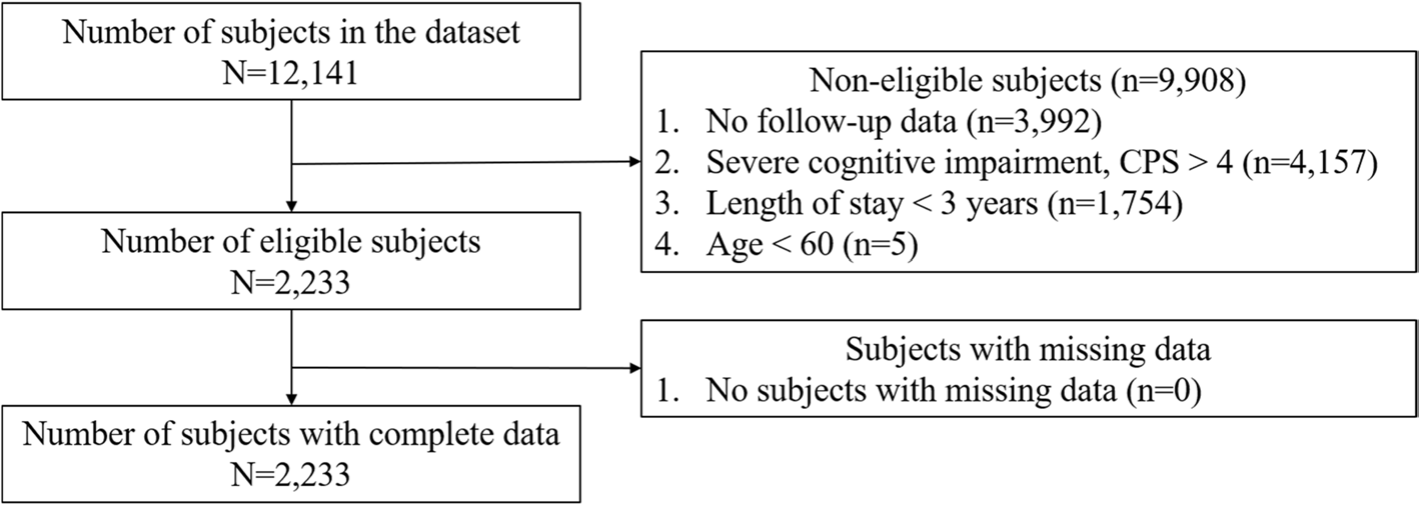
Subject selection flow chart

As shown in Fig. 1, there were 12,141 residents in 7 LTCFs in the dataset. Of these, 9,908 residents were not eligible for inclusion in this study, either because they had no follow-up data (*n* = 3,992), had severe cognitive impairment at baseline (*n* = 4,157), had stayed in the facilities for less than 3 years (*n* = 1,754), or were younger than 60 (*n* = 5). After the exclusion of non-eligible residents, 2,233 were left. All these residents had complete data on every variable in our analysis.

### Participants’ descriptive data

As shown in Table 1, the mean age of the residents was 82.1 (8.2) years. More residents were female (*n* = 1,530, 68.5%). The mean Cognitive Performance Scale score at baseline was 1.5 (SD = 1.2); the mean follow-up year was 4.4 (SD = 0.8) years. Most co-morbidities, except hypertension (*n* = 1,373, 61.5%), characterized only a small number of people, including dementia (*n* = 643, 28.8%), stroke (*n* = 684, 30.6%), and diabetes (*n* = 563, 25.2%). A relatively smaller portion of residents had hearing impairment (*n* = 881, 39.5%), visual impairment (*n* = 338, 17.4%), or dual sensory impairment (*n* = 273, 12.2%). Most residents used no hearing aids (*n* = 2,129, 95.3%) and most residents were not using visual aids (*n* = 1,620, 72.5%) at baseline. The mean decline in the Cognitive Performance Scale score from T0 to T1 was 0.4 (SD = 0.8).

**Table 1:** Demographics, predictors, and outcomes

Variables	*N* = 2,233
***Demographics and related clinical profile***	
Age, mean (SD)	82.1 (8.2)
Gender, n (%)	
Male	703 (31.5)
Female	1,530 (68.5)
Cognitive function at baseline (CPS), mean (SD)	1.5 (1.2)
Follow-up year, mean (SD)	4.4 (0.8)
Co-morbidities	
Dementia, n (%)	643 (28.8)
Stroke, n (%)	684 (30.6)
Diabetes mellitus, n (%)	563 (25.2)
Hypertension, n (%)	1,373 (61.5)
***Predictors***	
Hearing impairment, n (%)	881 (39.5)
Visual impairment, n (%)	388 (17.4)
Dual sensory impairment, n (%)	
No	1,237 (55.4)
Single	723 (32.4)
Dual	273 (12.2)
***Mediators***	
Hearing aid use pattern, n (%)	
Having no/ Not using hearing aids	2,129 (95.3)
Having hearing aids but not regularly used	56 (2.5)
Having hearing aids and often used	48 (2.1)
Visual aid use, n (%)	
Not using visual aids	1,620 (72.5)
Using visual aids	613 (27.5)
***Outcome***	
Change in cognitive function (CPS), mean (SD)	0.4 (0.8)

^CPS Cognitive Performance Scale^

As shown in Table 2, residents with visual impairment were mostly not using visual aids (*n* = 333, 14.9%) than those who were using visual aids (*n* = 55, 2.5%, *p* < 0.001). Residents with hearing impairment, the majority of them were not using hearing aids (*n* = 787, 35.2%), a small group of them used hearing aids (*n* = 43, 1.9%), and another small group of them had hearing aids but they did not use regularly (*n* = 51, 2.3%, *p* < 0.001).

**Table 2:** Crosstab between sensory impairment and use of aids

*n* (%)	Visual aids				*P*-value
Visual Impairment^a^	Yes		No		< 0.001
Yes	55 (2.5)		333 (14.9)		
No	558 (25.0)		1,287 (57.6)		
n (%)	Hearing aids				
Hearing Impairment^b^	Present and used	Present and not used regularly		No/not using	< 0.001
Yes	43 (1.9)	51 (2.3)		787 (35.2)	
No	5 (0.2)	5 (0.2)		1,342 (60.1)	

^a^Visual impairment was measured by a 5−point scale from MDS−RAI 2.0, with a score of 0–1 indicating no visual impairment

^b^Hearing impairment was measured by a 4−point scale from MDS−RAI2.0, with a score of 0 indicating no hearing impairment

### Main results

For hypothesis #1, as shown in Table 3, compared with those who had visual impairment, those who had no visual impairment had less cognitive decline (mean difference in CPS change score = -0.142, *p* = 0.004). Compared with those who had hearing impairment, those who did not have hearing impairment had less cognitive decline (mean difference in CPS change score = -0.083, *p* = 0.022). Compared with those who had a dual sensory impairment, those who had single sensory impairment (mean difference in CPS change score = -0.139, *p* = 0.021) and those who had no sensory impairment (mean difference in CPS change score = -0.205, *p* = 0.001) had a lower level of cognitive decline.

**Table 3:** Associations between visual impairment, hearing impairment, and dual sensory impairment with cognitive decline

Model	Predictors	Estimated marginal mean difference in CPS^a^ change score	F	*p*-value
1	Visual impairment		8.426	0.004
	No	-0.142		
	Yes	0		
2	Hearing impairment		5.237	0.022
	No	-0.083		
	Yes	0		
3	Dual sensory impairment		6.083	0.002 0.001 0.021
	No	-0.205		
	Single	-0.139		
	Dual	0		

^a^*CPS *cognitive performance scale

All models are adjusted for age, gender, and baseline cognitive function, dementia, diabetes, hypertension, and stroke

**Fig. 2:**
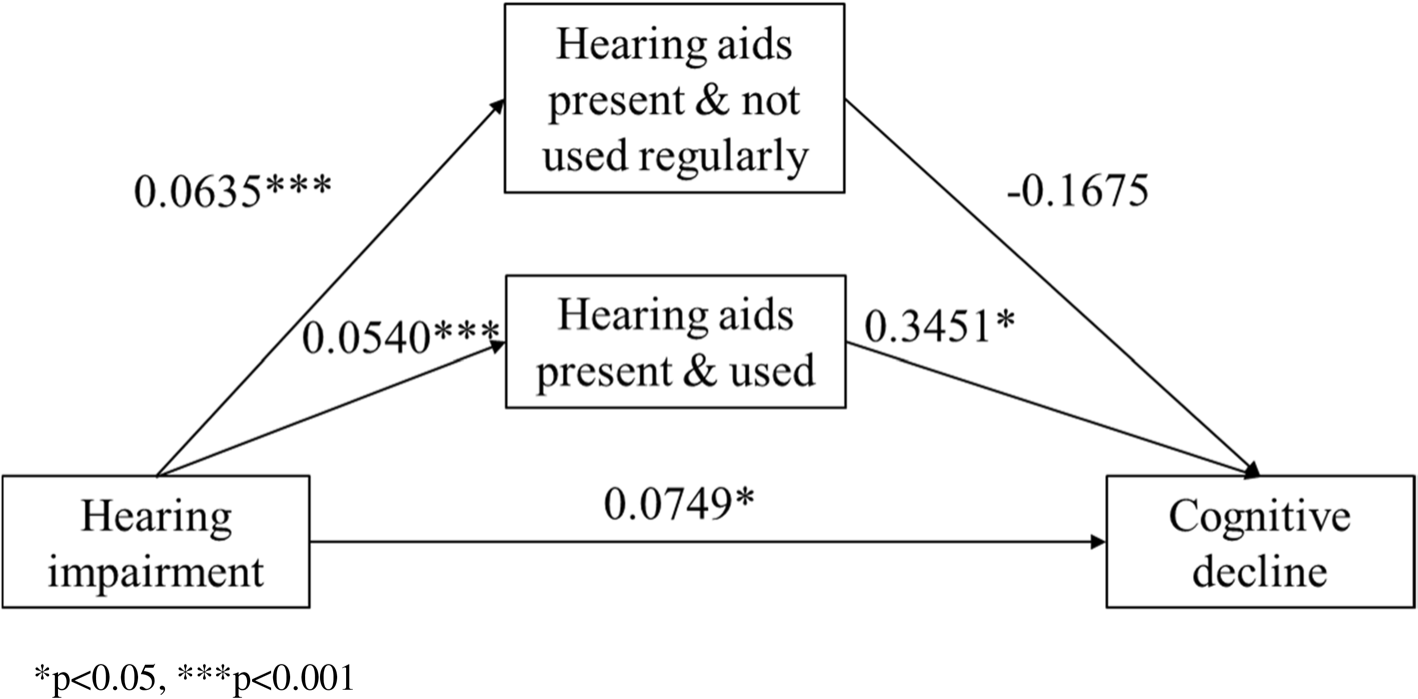
Mediation effects of the use of hearing aids on hearing impairment and cognitive decline

**Fig. 3:**
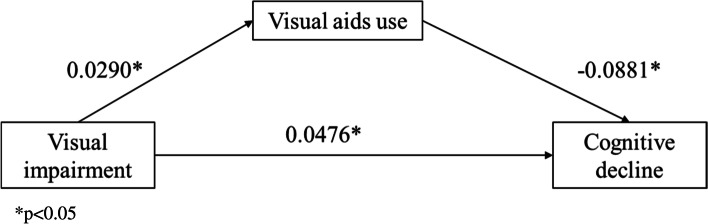
Mediation effects of the use of visual aids on visual impairment and cognitive decline

For hypothesis #2, as shown in Table 4 and Fig. 2, the total effect (coefficient = 0.0829) and direct effect (coefficient = 0.0749) between hearing impairment and cognitive decline were statistically significant. The indirect effect of a pattern of hearing aid use (i.e., hearing aids present and used) is statistically significant (coefficient = 0.0186). However, the indirect effect of another pattern of hearing aids use was not statistically significant. These findings showed that hearing impairment is independently associated with cognitive decline (direct effect, coefficient = 0.0749) and hearing impairment together with patterns of hearing aids use are associated with cognitive decline (total effect, coefficient = 0.0829). The effect of hearing impairment on cognitive decline is only positively mediated by the pattern of hearing aids present and used (indirect effect, coefficient of “hearing aids present and used” = 0.0186). As shown in Table 5 and Fig. 3, the total effect (coefficient = 0.0290), direct effect (coefficient = 0.0476), and indirect effect of visual aids use (coefficient = -0.0026) were all statistically significant. These findings showed that visual impairment is independently associated with cognitive decline (direct effect, coefficient = 0.0476) and visual impairment together with the use of visual aids are associated with cognitive decline (total effect, coefficient = 0.0290). The effect of visual impairment on cognitive decline is negatively mediated by the use of visual aids (indirect effect of visual aids use, coefficient = -0.0026).

**Table 4:** Total, direct and indirect effects of hearing impairment on cognitive decline

	Effect coefficient
Total	0.0829*
Direct	0.0749*
Indirect	
Hearing aids present but not regularly used	-0.0106
Hearing aids present and used	0.0186*

*p<0.05

**Table 5:** Total, direct and indirect effects of visual impairment on cognitive decline

	Effect coefficient
Total	0.0450*
Direct	0.0476*
Indirect	
Use of visual aids	-0.0026*

*p<0.05

## Discussion

To the authors’ best knowledge, this is one of the very few studies demonstrating the protective effect of visual aids on cognitive decline in LTCFs. It is also among the very few to illustrate the effect of hearing aid use patterns on cognitive decline in LTCFs. In terms of strength, the data in this study were collected by highly trained and accredited healthcare professional assessors in multiple sites in Hong Kong, and there were no missing data for any variable. The key findings of this study are multiple. First, hearing impairment is associated with a higher risk of cognitive decline and the hearing aids users have the worst hearing impairment and higher risk of cognitive decline. Second, visual impairment is associated with a higher risk of cognitive decline and visual aids users have a reduced risk of cognitive decline. We further explained the findings below.

Residents who often used hearing aids had the most severe hearing impairment. This pattern conveys that in the group of residents who used hearing aids often may still have not adequate restoration of hearing function. This could be because the hearing impairment in this group is caused by central hearing loss which could not be improved with peripheral amplification, such as hearing aids [58]. Another possible reason is that appropriate fitting was not provided to solve the common problems of hearing aids faced by the resident, such as noise interference [59]. Unfortunately, no data were collected to inform this issue. It is known that there is a progressive hearing loss along with age and the hearing loss in the 10^th^ decade of life becomes much more significant (i.e., 3.2–3.8 dB hearing loss per year) [60]. Without regular fittings and assessment of patterns of use, the protective effects of hearing aids decline over time. Hearing aid fitting for older residents in this set of LTCFs was not documented and pertinent data were not available in the MDS. Evidence shows that regular hearing aid fitting improves the hearing function of older people and the quality of life of the significant others [61]. Therefore, this study recommends diagnosing causes of hearing loss for LTCF residents, regular fit check, and routine monitoring of fit and use to maximize the hearing restorative effects of the hearing aids.

In the literature, reported hearing aids use was associated with slowing down the cognitive decline of older people with hearing impairment in community settings [62]. A hearing aid is promoted as a potentially important measure to prevent dementia by rectifying the hearing loss [63]. However, in this study, hearing aids use was associated with a higher risk of cognitive decline. This is probably because the protective effect of the hearing aids against cognitive decline only happens when hearing aids could improve audibility [64]. In Hong Kong, audibility tests (e.g., pure-tone audiometry) was not regularly conducted among LTCF residents to identify whether their hearing aids were effective to promote hearing function. We recommend that the visiting primary doctor includes or makes referrals for an audibility test (with and without hearing aids) in the regular medical follow-up and assessment for all the LTCF residents.

Visual impairment is associated with an increased risk of cognitive decline and the use of visual aids has the potential to protect residents from cognitive decline. This finding is consistent with previous literature [65]. The sensory loss consequence theory explains that sensory impairment reduces the ability of older people to participate in activities, which may decrease brain stimulation and neural reorganization, and increase social isolation, subsequently leading to cognitive decline [66]. Having better sensory function may prompt older people to take on more cognitively stimulating tasks to reduce the risk of neurodegeneration [39]. Another finding in this study was that the residents who were not using visual aids had more severe visual impairment. This implies that visual aids in this population are under-used. Therefore, this study recommends that regular screening for visual impairment followed by visual aids prescription should take place in LTCF residents to correct their visual function at an earlier stage of visual impairment in order to reduce their risk of cognitive decline. Further studies should examine why the LTCF residents with visual impairment did not use visual aids.

Dual sensory impairment is regarded as a risk factor of cognitive impairment [67]. Compared with the residents with single sensory impairment, those with dual sensory impairments usually experience more difficulty in daily activities resulting in a greater reduction in physical activities and social interaction [68]. This may explain our findings showing that the residents with dual sensory impairment had more severe cognitive decline, compared with those who had a single sensory impairment and those who had no sensory impairment. This finding also aligned with previous studies indicating that more attention should be paid to the residents with dual sensory impairment [28]. Perhaps for LTCF residents, assessment of visual and hearing ability is as important as the classic vital signs of blood pressure, pulse, respirations and pain.

### Limitations

There are several limitations to this study. First, sensory function was assessed by allowing the residents to see or hear with the aids that they commonly used. Therefore, this measurement did not reflect their capacity. Instead, it was the performance after considering their pattern of utilization of sensory aids. Second, sampling bias was probable since the median length of stay in the LTCF was only 73.4 weeks (i.e., 1.4 years) [52]. Therefore, the majority of the residents were excluded because they were not eligible (i.e., 81.6%). Third, sensory impairment, the use of audio-visual aids and the confounders of sensory impairment on the cognitive decline were observed at baseline only which was after 3 years of residency. The progression of sensory impairment and the presence of confounding factors for the cognitive decline throughout longitudinal observation is not known. Fourth, the cognitive function was measured by a 7-point Cognitive Performance Scale that may not be sensitive enough to identify minute but important changes in cognitive decline. It is because a validation study on CPS showed that CPS explained only 48.8% of the variability in MMSE, although it has a satisfactory diagnostic accuracy on cognitive impairment (area under curve = 0.73) [69]. Fifth, the use pattern of audio-visual aids was assessed once in 6–12 months by asking the participants or their caregivers in the LTCF. The internal validity may be threatened by recall bias. Lastly, without information about the hearing aid or visual aid fitting and function, the cause of impairment could not be clear.

## Conclusion

To prevent cognitive decline, this study highlighted the importance of providing regular screenings for visual impairment with subsequent visual aids prescription and monitoring of use and function. To ensure hearing aids could improve hearing function, regular objective audibility tests with and without hearing aids and regular hearing aid fitting should be conducted among LTCF residents. Further studies should incorporate hearing aid fitting and objectively assessed hearing function to examine whether hearing aids could restore residents’ audibility adequately. Also, further studies should examine why the LTCF residents with visual impairment did not use visual aids. Routine screening and monitoring of visual and auditory impairment and resident function might potentially change their quality of life and caregiver burden.

## Data Availability

The datasets generated and analysed during the current study are not publicly available due to the restrictions of our ethical approval which states that the data would only be shared within the research team, but are available from the corresponding author on reasonable request.

## Abbreviations

CPS: Cognitive Performance Scale
MDS: Minimum Data Set
LTCF: Long-term care facilities
